# Emergence of Super Cooperation of Prisoner’s Dilemma Games on Scale-Free Networks

**DOI:** 10.1371/journal.pone.0116429

**Published:** 2015-02-02

**Authors:** Angsheng Li, Xi Yong

**Affiliations:** 1 State Key Laboratory of Computer Science, Institute of Software, Chinese Academy of Sciences, Beijing, P. R. China; 2 University of Chinese Academy of Science, Beijing, P. R. China; Shanxi University, CHINA

## Abstract

Recently, the authors proposed a quantum prisoner’s dilemma game based on the spatial game of Nowak and May, and showed that the game can be played classically. By using this idea, we proposed three generalized prisoner’s dilemma (GPD, for short) games based on the weak Prisoner’s dilemma game, the full prisoner’s dilemma game and the normalized Prisoner’s dilemma game, written by GPD_W_, GPD_F_ and GPD_N_ respectively. Our games consist of two players, each of which has three strategies: cooperator (*C*), defector (*D*) and super cooperator (denoted by *Q*), and have a parameter *γ* to measure the entangled relationship between the two players. We found that our generalised prisoner’s dilemma games have new Nash equilibrium principles, that entanglement is the principle of emergence and convergence (i.e., guaranteed emergence) of super cooperation in evolutions of our generalised prisoner’s dilemma games on scale-free networks, that entanglement provides a threshold for a phase transition of super cooperation in evolutions of our generalised prisoner’s dilemma games on scale-free networks, that the role of heterogeneity of the scale-free networks in cooperations and super cooperations is very limited, and that well-defined structures of scale-free networks allow coexistence of cooperators and super cooperators in the evolutions of the weak version of our generalised prisoner’s dilemma games.

## Introduction

The prisoner’s dilemma (PD, for short) game is one of the well-known games, having implications in a wide range of disciplines. In a PD game, two players simultaneously decide their strategy *C* (cooperator) or *D* (defector). For mutual cooperation, both players receive a reward *R* and receive punishment *P* upon mutual defection. If one cooperates and the other defects, then the cooperator gains the lowest payoff *S* and the traitor gains temptation *T*. The payoff rank for the PD game is given by *T* > *R* > *P* > *S*.

The normalized version of the prisoner’s dilemma game is usually defined by a parameter *r*, in which case, the reward *R* = 1, the temptation *T* = 1 + *r*, the punishment *P* = 0 and the lowest sucker’s payoff *S* = −*r*.

Nowak and May [1] introduced the weak version of the prisoner’s dilemma game, in which the payoffs are chosen as *R* = 1, *P* = *S* = 0, and *T* = *b* for *b* > 1.

In a PD game, the best strategy for both players is to defect regardless of the other’s decision, in which case, the payoffs of the players are minimized.

Real games are played in a system characterized by a graph in which the nodes are players, and the edges represent the games played by the two endpoints of each of the edges. The games in a graph are evolving by rounds. In this case, emergence of cooperation of the evolutionary games on a graph implies a maximal global payoff evolved from minimal local payoffs of the graphs. It plays an essential role in the organizations of the graphs such as systems of biosphere and human society. Evolutionary game theory is devoted to understanding the emergence of cooperation and the cooperative behaviors in the games in nature and society.

Nowak and May [1] proposed a weak version of the prisoner’s dilemma game and explored that spatial reciprocity (or spatial structure) is a mechanism promoting cooperation. Social structures are fund to play a role in emergence of cooperations in evolutionary games, referred to [2–4]. It was recognised long time ago that heterogeneity of networks plays a role in the emergence of cooperations of evolutionary games in networks [5].

Santos and Pacheco showed that the heterogeneity of scale-free networks promotes emergence of cooperation [6–8]. All these results showed that heterogeneity does promote emergence of cooperation in different games. This property has also been found in other games, for instance, in the public goods games (PGG) [10]. On the other hand, cooperation is unlikely to emerge in the evolutionary prisoner’s dilemma games on homogeneous networks, referred to [11–13].

Pacheco and Santos [14] reported that cooperations emerge around the largest hub. This property has also been found in other games [7, 9, 10, 12, 15–17], which further demonstrates that heterogeneity of networks, a metaphor for social diversity, favors the emergence of cooperative behavior.

Wu et al. [18] studied the roles of different choices of updating strategies in the emergence of cooperation in evolutionary prisoner’s dilemma games on the scale-free networks of the PA model [6], Rong and Wu [19] investigated the role of degree correlation in public goods games on the scale-free networks. Rong, Wu and Chen [20] proposed a normalised payoff model of the spatial prisoner’s dilemma game, with which cooperation fails to emerge in the evolutions of the weak prisoner’s dilemma games on the scale-free networks.

Wang, Szolnoki and Perc studied the role and limitation of the reciprocity in the cooperative behaviors of a network from external and independent networks [21, 22], and the role of rewarding fitness of links in the formation of cooperations in evolutionary games [23]. Chen, Szolnoki and Perc [24] proposed a model for sharing the cost of punishment in evolutionary games.

Eisert *et al* in 1999 [25] proposed a quantum game in which each player chooses a strategy from a unitary operator. Li *et al* [26–28] implemented some experiments on evolutionary quantum games on some networks, in which each player randomly chooses a strategy from the whole unitary space. The experiments showed that quantum strategies promote cooperations in the games for some choices of the strategies. In particular, for two special quantum strategies, the Hadamard operation and the *Q* strategy (the same as our super cooperator), the experiments in [27] showed that the grid network is most easily invaded by quantum strategies and that a scale-free network can be invaded by agents adopting quantum strategies only if a hub of the network is occupied by an agent with a quantum strategy or if the fraction of agents with quantum strategies in the population is significantly large. Pawela and Sladkowski [29] investigated the evolution of strategies on hyper-graph networks with three quantum strategies. The experiments in [29] showed that in the case of a three player game on a hyper-graph networks, quantum strategies are not necessarily stable strategies, and that in some cases, the defection strategy is as good as a quantum strategy. Chappell, Iqbal and Abbott [30] proposed an analysis of three-player quantum games in an EPR type setup by using the Clifford geometric algebra.

Nowak [31] proposed five simple rules for cooperation, including: kinship, direct reciprocity, indirect reciprocity, spatial structure and group selection etc. Li and Yong [32] proposed a quantum prisoner’s dilemma game by introducing entanglement in the games and by introducing a new strategy of super cooperator in the prisoner’s dilemma games. It was shown that super cooperation is guaranteed to quickly emerge in the evolutionary quantum prisoner’s dilemma games on random graphs generated by the Erdös-Rényi model [33], and the small world model [34].

By using the ideas in [32], we will propose three versions of generalised prisoner’s dilemma games, and investigate the emergence and convergence (or guaranteed emergence) of (super) cooperations in evolutionary games on scale-free networks of the PA model [6].

## Results

We proposed three generalized prisoner’s dilemma (GPD, for short) games based on the weak prisoner’s dilemma game, the full prisoner’s dilemma game and the normalized prisoner’s dilemma game, written by GPD_W_, GPD_F_ and GPD_N_ respectively. Our games consist of two players, each of which has three strategies: cooperator (*C*), defector (*D*) and super cooperator (denoted by *Q*), and have a parameter *γ* to measure the entangled relationship between the two players. We found that our games have a new Nash equilibrium principle, and that entanglement is the principle of emergence and convergence (that is, guaranteed emergence) of super cooperation. Our experiments demonstrate that for every temptation *b*, and for appropriately large entanglement *γ*, either super cooperation emerges or cooperation and super cooperation coexist and dominate the network in the evolutionary GPD_W_ games, that for every benefit-cost ratio *r* in the normalised prisoner’s dilemma game, there is a phase transition for the entanglement *γ* at some threshold *γ*_0_ for evolutionary GPD_N_ games, that for the evolutionary GPD_W_ games with normalised payoff updating strategy, there is an interval (*γ*_0_, *γ*_1_) such that if the entanglement *γ* ≤ *γ*_0_, then defection dominates the network, and if *γ* ≥ *γ*_1_, then super cooperation dominates the network, and that the normalised payoff updating strategy plays no role in the evolutionary GPD_N_ games on the scale-free networks.

Our generalized prisoner’s dilemma games are natural extensions and generalizations of the classical prisoner’s dilemma (PD, for short) game. The Nash equilibrium principles of the GPD’s solve the prisoner’s dilemma. The new principles of our convergence of super cooperation, destroyer elimination, emergence of super cooperation and the coexistence of cooperation and super cooperation, and phase transition phenomena of super cooperation provide an approach to solving the prisoner’s dilemma in evolutions of prisoner’s dilemma games on arbitrarily given complex networks. Our games and principles explore that entanglement is the mechanism of emergence and convergence of cooperation in a rich-get-richer world, that entanglement provides a phase transition from the domination of defectors to the domination of super cooperators, and that in evolutions of the weak version of our generalised prisoner’s dilemma games on the scale-free networks with well-defined structures, cooperation and super cooperation may coexist, and jointly dominate the networks. Our theory demonstrates that entanglement is the mechanism of guaranteed emergence of super cooperation in arbitrarily given networks, for arbitrarily large temptations and benefit-cost ratios with any reasonable updating strategy, that entanglement provides a threshold for a phase transition of emergence of super cooperation in evolutions of the normalised version of our generalised prisoner’s dilemma games on scale-free networks, that the role of heterogeneity in emergence of cooperation and super cooperation is very limited in evolutions of both the classical and generalised prisoner’s dilemma games, and that well-defined structures of networks allow coexistence of cooperation and super cooperation in evolution of the weak generalised prisoner’s dilemma games, in which case, the collection of cooperators and super cooperators dominate the networks.

### Generalized Prisoner’s Dilemma Games

We propose our *generalized prisoner’s dilemma* (GPD, for short) games based on quantum mechanics. Our idea is to introduce a special strategy, written by *Q*, which joins the classical prisoner’s dilemma game as a *super cooperator*. Therefore, our game consists of three strategies: *C*, *D* and *Q*, given by $C=\hat{U}(0,0)$, $D=\hat{U}(\pi ,0)$, and $Q=\hat{U}(0,\frac{\pi }{2})$, where *U* is the unitary operator, defined by:

$$\hat{U}\left(\theta ,\phi \right)=\left(\begin{array}{cc} e^{i\phi }\cos\theta /2 & \sin\theta /2\\ -\sin\theta /2 & e^{-i\phi }\cos\theta /2 \end{array}\right),$$

for *θ* and *ϕ* ranging from 0 to *π* and from 0 to $\frac{\pi }{2}$ respectively.

Our game consists of two players, Alice and Bob. We assume that Alice and Bob have a measurement of entanglement $\gamma \in [0,\frac{\pi }{2}]$, and that if both Alice and Bob choose strategies *C* or *D*, then the game between Alice and Bob is the same as a classical prisoner’s dilemma game. Based on these assumptions, we build a game between Alice and Bob by quantum mechanics, which is hence a natural extension of the classical prisoner’s dilemma game.

In the real world game between two players, Alice and Bob say, there is always a complicated relationship, including many factors such as friendship, kinship, trust, confidence etc. In our theory, we use an entanglement degree *γ* to represent the mixture of all the possible relationships between the two players.

There are three versions of our generalised prisoner’a dilemma games. They are the weak version, the full version and the normalised version, which are built based on the spatial game of Nowak and May, the full prisoner’s dilemma game, and the normalised prisoner’s dilemma game respectively.

### The Weak Version

The weak version of our generalised prisoner’s dilemma game is the same as that in [32]. We use GPD_W_ to denote the game. The payoff matrix of this generalized prisoner’s dilemma game is thus given by Table 1 below.

**Table 1:** GPD_W_. The expectation payoff matrix of our generalized prisoner’s dilemma game—the weak version

	***C***	***D***	***Q***
*C*	(1, 1)	(0, *b*)	(cos^2^ *γ*, cos^2^ *γ*)
*D*	(*b*, 0)	(0, 0)	(*b* ∙ cos^2^ *γ*, *b* ∙ sin^2^ *γ*)
*Q*	(cos^2^ *γ*, cos^2^ *γ*)	(*b* ∙ sin^2^ *γ*, *b* ∙ cos^2^ *γ*)	(1, 1)

In our game, we choose just one quantum strategy, $Q=\hat{U}(0,\frac{\pi }{2})$, and allow the measurement of entangled relationship *γ* to change from 0 to $\frac{\pi }{2}$. We notice that the payoff matrix is deduced from quantum mechanics, but the game itself can be simulated classically. By this reason, our generalized prisoner’s dilemma is a game based on quantum mechanics that can be played by both quantum and classic devices. However the significance of our generalized prisoner’s dilemma game is to solve the classical prisoner’s dilemma in real world games. Notice that all the games in the experiments of our paper are played classically.

From Table 1, we have the following results:

1) If *γ* = 0, then the *Q* strategy collapses at *C*;
2) If both players choose the strategy *Q*, then they will get payoff 1, the same as that they both choose the strategy *C*, and *Q* never takes advantage from *C*;
3) If $\gamma =\frac{\pi }{2}$, then a (*Q*, *C*) game gets payoffs (0,0) respectively, and if $\gamma =\frac{\pi }{2}$, then a (*Q*, *D*) game gets payoffs *b* and 0 respectively, meaning that *Q* takes advantage from *D*, and if *γ* > 0, then *Q* always reduces the gain of a *D* strategy; and
4) when *γ* increases, (*Q*, *Q*) becomes more and more a stable game than the (*C*, *C*) game due to the entanglement of the two players, and if $\gamma =\frac{\pi }{2}$, then (*Q*, *Q*) is a Nash equilibrium that maximizes the payoffs.

By 1), the classical prisoner’s dilemma game is a special case of our game. By 2), we can interpret *Q* as a cooperator. By 4), if $\gamma =\frac{\pi }{2}$, then *Q* is completely different from *C*, meaning that *Q* is indeed a new strategy. By 3), *Q* is against *D*. By 4), the game (*Q*, *Q*) would be a Nash Equilibrium, and simultaneously be Pareto optimal. Therefore, our game is a natural generalization of the prisoner’s dilemma with a new strategy, the super cooperator *Q*.

The motivations of our *Q*-strategy and the GPD game are as follows:

(1) The *Q*- strategy and the generalized prisoner’s dilemma game capture new phenomena in nature and society. In biological species evolution, initially we have a population *C*, later on, a species of invaders *D* appears, the invaders take advantage from the population *C*. In this case, there will be a new type mutated from population *C*, which is the *Q*-strategy. The new type *Q* is consistent with *C*, but is against the invaders *D*. Eventually, the new type *Q* will evolve as a new dominating species. In human society, we consider the honest crowds as *C*, and dishonest crowds as *D*. To prevent the invasions of the *C*-strategy crowds from the *D*-strategy invaders, a party *Q* naturally and gradually grows up from *C* to fight with *D* so that the *D*-strategy invaders become less and less in a society.
(2) The generalized prisoner’s dilemma game may explain some new phenomena in economy. For instance: it explains the reason why a win-win strategy in economics is possible, and it provides a mechanism to guarantee such a win-win strategy. By the GPD, we know that the reason why a win-win cooperation is possible in a prisoner’s dilemma game is that the two players in the game have some entangled relationship.
(3) The generalized prisoner’s dilemma game may explain the games among countries in international relations. It is interesting to notice that our GPD would explain the evolutionary games among different countries. For instance, in the games between China and US, it seems hard for either side to take a firm *D* strategy due to the deeply entangled relationship between the two countries. Therefore the GPD is certainly a new type of games with both theoretical and practical implications.
(4) Entanglement is essential to games, for which our GPD fully capture. Remarkably, our GPD fully explores the roles and principles of entanglement between the players in the prisoner’s dilemma game. This makes our game completely different from the classic prisoner’s dilemma game.

Remarkably, our weak GPD has a new Nash equilibrium principle. Given a temptation *b* ∈ (1, 2) and $\gamma \in [0,\frac{\pi }{2}]$, suppose that Alice and Bob play the game with payoff matrix given in Table 1.

Suppose that $\gamma >\arccos\frac{\sqrt{b}}{b}$. We consider the following scenario: If Alice chooses a *Q*-strategy, then the maximal payoff of Bob is max{1, cos^2^ *γ*, *b* ∙ cos^2^ *γ*}. By the assumption of $\gamma >\arccos\frac{\sqrt{b}}{b}$, the best payoff of Bob max{1, cos^2^ *γ*, *b* ∙ cos^2^ *γ*} is minimized at 1, which occurs only if Bob chooses *Q* too. Therefore, if $\gamma >\arccos\frac{\sqrt{b}}{b}$, then by choosing *Q*, Alice forces Bob to choose *Q* if Bob wants to maximize her payoff. At the same time, when both Alice and Bob choose *Q*, the payoffs of both players and the total game are maximized. By the same reason, in this case, (*Q*, *Q*) is the unique Nash equilibrium of our weak generalized prisoner’s dilemma game.

The result above explores a new principle of the generalized prisoner’s dilemma game.

*Nash equilibrium principle of the generalised prisoner’s dilemma game—weak version*: If $\gamma >\arccos\frac{\sqrt{b}}{b}$, then a player may force its opponent to cooperate by choosing the strategy *Q*.

Clearly the phenomenon explored above frequently occurs in real world games, but never in classical prisoner’s dilemma games. Our result implies that entangled relationship between the two players maybe the reason and mechanism of a win-win cooperation in a prisoner’s dilemma game in real world.

### The Full Version

Suppose that two players Alice and Bob have an entanglement measured by *γ*, and that Alice and Bob are playing a full version of prisoner’s dilemma game. Then by a quantum device, the payoff matrix of the game is given by Table 2. We say that this game is the full version of generalised prisoner’s dilemma game, and written by GPD_F_.

**Table 2:** GPD_F_. The expectation payoff matrix of our generalized prisoner’s dilemma game—the full version. The value in the matrix is the payoff of the row strategy.

	***C***	***D***	***Q***
*C*	*R*	*S*	*R* ∙ cos^2^ *γ* + *P* ∙ sin^2^ *γ*
*D*	*T*	*P*	*T* ∙ cos^2^ *γ* + *S* ∙ sin^2^ *γ*
*Q*	*R* ∙ cos^2^ *γ* + *P* ∙ sin^2^ *γ*	*S* ∙ cos^2^ *γ* + *T* ∙ sin^2^ *γ*	*R*

From Table 2, if the entanglement degree *γ* = 0, then the super cooperator collapses to the cooperator, and the full version of generalised prisoner’s dilemma game is identical to the classical prisoner’s dilemma game. As the entanglement degree *γ* increases, the super cooperator *Q* takes more and more advantage from the defector *D*.

Suppose that Alice and Bob play a game with payoff matrix given in Table 2. If Alice chooses a *Q*-strategy, then the maximal payoff of Bob is max_Bob_ = max{*R* ∙ cos^2^ γ + *P* ∙ sin^2^ *γ*, *T* ∙ cos^2^ *γ* + *S* ∙ sin^2^ *γ*, *R*}. Assume that $\gamma >\arccos\sqrt{\frac{R-S}{T-S}}$. Then Bob achieves its maximal payoff only if it chooses a *Q*-strategy.

Therefore, the generalised prisoner’s dilemma game satisfies the following principle.

*Nash Equilibrium principle of the generalised prisoner’s dilemma game—full version*. If $\gamma >\arccos\sqrt{\frac{R-S}{T-S}}$, then (*Q*, *Q*) is the unique Nash equilibrium which achieves the maximal payoffs.

Clearly, the Nash equilibrium principle for the weak version of the generalised prisoner’s dilemma game is a consequence of the principle above.

### The Normalised Version

By Table 2, we can easily give the normalised version of generalised prisoner’s dilemma game. We describe this normalised version in Table 3, and use GPD_N_ to denote the game.

**Table 3:** GPD_N_. The expectation payoff matrix of our generalized prisoner’s dilemma game—the normalised version. The value in the matrix is the payoff of the row strategy.

	***C***	***D***	***Q***
*C*	1	−*r*	cos^2^ *γ*
*D*	1 + *r*	0	(1 + *r*) ∙ cos^2^ *γ* − *r* ∙ sin^2^ *γ*
*Q*	cos^2^ *γ*	−*r* ∙ cos^2^ *γ* + (1 + *r*) ∙ sin^2^ *γ*	1

For the game in Table 3, we have:

*Nash Equilibrium principle of the generalised prisoner’s dilemma game—normalised version*. If $\gamma >\arccos\sqrt{\frac{1+r}{1+2r}}$, then (*Q*, *Q*) is the unique Nash equilibrium which achieves the maximal payoffs.

In the present paper, we investigate the role of entanglement degree *γ* in the emergence and convergence (i.e., guaranteed emergence) of (super) cooperation in the evolutions of the prisoner’s dilemma (PD), weak, and normalised versions of our generalised prisoner’s dilemma games, i.e., GPD_W_ and GPD_N_, on scale-free networks of the PA model [6].

How can we guarantee the emergence of cooperation or super cooperation of evolutionary generalized prisoner’s dilemma games on scale-free networks? We will answer this question. For this, we first look at the problems of the evolutions of classical prisoner’s dilemma games on some scale-free graphs.

### Divergence of Cooperations of Classic Prisoner’s Dilemma Games on Scale-free Networks

Barabási and Albert [6] proposed a model of networks by introducing preferential attachment (denoted by PA, for short) as an explicit mechanism such that the networks generated by the model follow a power law. As mentioned above, Santos and Pacheco [7] showed that the scale-free networks generated by the PA model provides a framework for emergence of cooperation. In fact, it has been shown that heterogeneity of graphs generated from the PA model promotes emergence of cooperations in evolutionary games on the graphs [7–9].

However, global cooperation of evolutionary prisoner’s dilemma games is hard to emerge on power law networks. It was known that cooperation fails to emerge in the evolutions of the full or normalised full version of prisoner’s dilemma games on scale-free networks of the PA model, and that even if for the weak version PD games, cooperation fails to emerge in the evolutions of the games with normalised payoff updating strategy [18].

We will see that even if for the weak prisoner’s dilemma games, emergence of cooperations in the evolutions of the games on scale-free networks has a number of limitations. To understand this, we will show that the equilibrium frequencies of cooperations of evolutionary prisoner’s dilemma games on graphs generated from the PA model diverge.

In the PA model, the only mechanism is the preferential attachment, it constructs networks by steps. Given a natural number *d*, and an initial graph *G*_0_. At step *t* + 1, let *G*_*t*_ be the graph constructed at the end of step *t*, create a new node, *v* say, and create *d* edges (*v*, *u*) for some *u*’s, where each *u* is chosen with probability proportional to the degrees of nodes in *G*_*t*_.

At first, we define the evolution of the weak prisoner’s dilemma games.

Let *G* = (*V*, *E*) be a network. Let *ε* be a constant in (0,1), and temptation *b* be a constant in (1,2). The evolutionary PD games in G proceeds as follows:

1) At step 0, for every node *v* ∈ *V*, let *s*(*v*)[0] be the strategy of *v* during step 0. With probability *ε*, define *s*(*v*)[0] = *C*, otherwise, then define *s*(*v*)[0] = *D*.
  For every *v* ∈ *V*, the payoff of *v* at step 0 is the total payoffs between *v* and all its neighbors in *G*. We use payoff(*v*)[0] to denote the payoff of node *v* at step 0.
2) At step *t* + 1, for every node *v* ∈ *V*, randomly and uniformly choose a neighbor, *u* say, of *v*, if payoff(*u*)[*t*] > payoff(*v*)[*t*], then with probability $\frac{P(u)[t]-P(v)[t]}{b∙\max\{d_{u},d_{v}\}}$, define *s*(*v*)[*t*+1] = *s*(*u*)[*t*], otherwise, then *s*(*v*)[*t* + 1] = *s*(*v*)[*t*].

For every *t*, define *ρ*(*G*)[*t*] to be the fraction of nodes *v*’s such that *s*(*v*)[*t*] = *C* in *V*. Notice that for every *t*, *ρ*(*G*)[*t*] is a random variable, depending on the random choices prescribed in the evolutionary PD games.

We say that cooperation emerges in the evolutionary PD games on *G*, if there are *t* such that the expectation of *ρ*(*G*)[*t*] is larger than a majority constant, $\frac{2}{3}$ say. That is: *E*[*ρ*(*G*)[*t*]] > *δ*, for some constant $\delta \geq \frac{2}{3}$.

Santos and Pacheco [7] numerically verified that cooperation may emerge in the evolutions of the weak PD games on some networks generated by the PA model with appropriate choices of parameters. However, there is no any analysis of guaranteed emergence of cooperations of evolutionary games on the graphs, that is, the emergence of cooperation fails to give us any information about the concentration of random variables *ρ*(*G*)[*t*]. We will see that on graphs generated from the PA model, it is unlikely to have any useful concentration result of the equilibrium frequency of cooperation of the evolutionary weak PD games.

**Fig 1:**
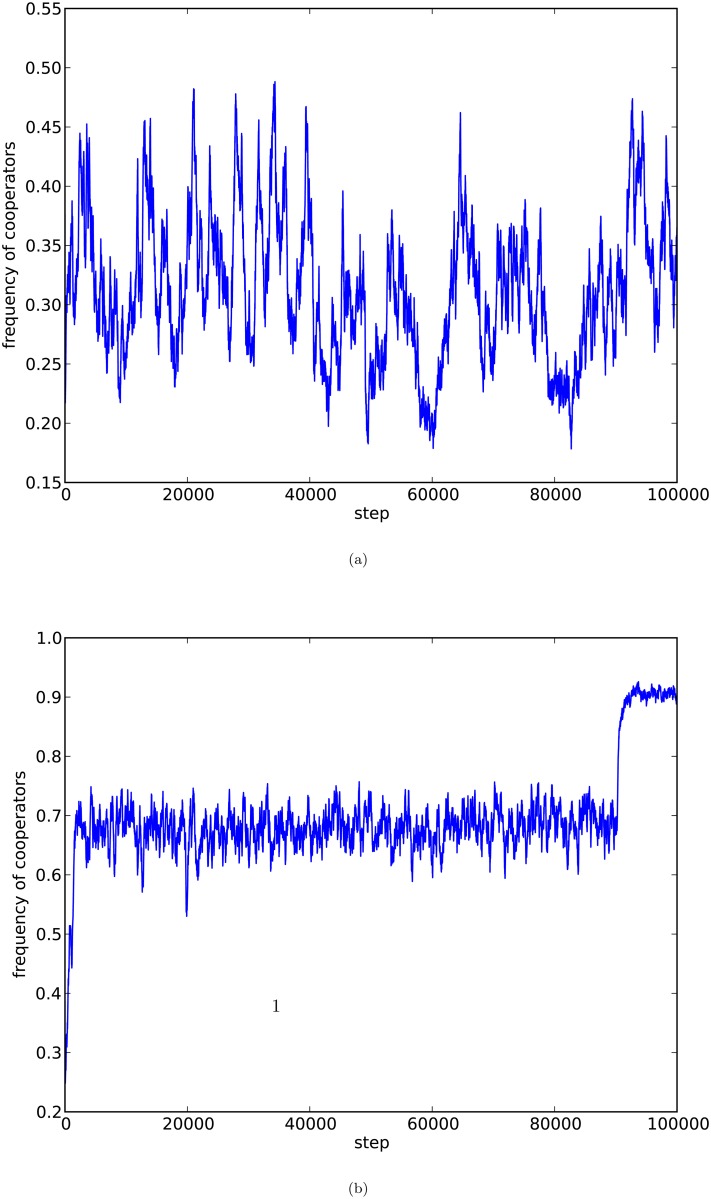
Weak prisoner’s dilemma, PA model. In both (a) and (b), we depict the frequency of cooperators of PD games with *ε* = 0.5 and *b* = 1.5 of a network of the PA model with number of nodes *n* = 10,000 and *d* = 4 with two networks each of which has one evolution of the weak PD games.

In Figs. 1 (a), and 1(b), we depict two curves of cooperation ratios of the weak prisoner’s dilemma games on a network of the PA model with *d* = 4, each curve corresponds to an evolution of the weak prisoner’s dilemma games.

From Fig. 1, we observe the following:

*Divergence of cooperation phenomenon*: The equilibrium frequencies of cooperations of the weak prisoner’s dilemma games on scale-free networks of the PA model fail to quickly emerge or fluctuate in a large interval.

Furthermore, the equilibrium frequencies of cooperations diverge in different ways such as: global cooperation may fail to emerge, or the frequencies fluctuate in a wide range, or emerges after a huge number of rounds of the games, or vanishes unexpectedly after even if it emerged. However the emergence of cooperation of evolutionary weak prisoner’s dilemma games on scale-free networks of the PA model, is unexpected, unpredictable, and un-interpretable, so that the emergence of cooperation is useless practically, even if it emerged.

We notice that time is an important factor for the emergence of cooperation of the games. The experiments above show that in the evolutionary weak prisoner’s dilemma games on the scale-free networks, even if cooperation emerges, it may take a huge number of rounds of the evolutions.

Our experiments in Fig. 1 clarifies the roles of heterogeneity in the emergence of cooperation as follows:

1) Cooperation emerges only statistically, or by chance, instead of guaranteed,
2) Cooperation fails to converge,
3) Cooperation fails to quickly emerge, even if it is going to emerge, and
4) Emergence of cooperations may varnishes suddenly without reasonable explanation.

The results in 1)–4) above indicate that the heterogeneity along plays only a very limited role in the formation of cooperative behaviors in the game of selfish individuals. We thus conjecture that there must be more fundamental mechanisms for cooperation in nature and society.

In fact, cooperation is unlikely to emerge on networks of the PA model for large *d*’s, this is the reason why most people implemented experiments with only small *d*’s, and our experiments in the remaining parts of this paper imply that cooperation is unlikely to emerge in general prisoner’s dilemma games, but only in the weak games by Nowak and May [1], and that the emergence of cooperations depends on specific choices of updating strategies in the evolutions of the games.

### Convergence of Super Cooperations of Generalized Prisoner’s Dilemma Games on Scale-free Graphs

Let us consider the evolutions of the weak version of our generalized prisoner’s dilemma games given in Table 1 on the scale-free networks generated by the PA model. The weak version of our generalized prisoner’s dilemma game can be played on networks by the same way as that of the classic prisoner’s dilemma game.

Let *G* = (*V*, *E*) be a graph, and *p*_1_, *p*_2_ and *p*_3_ be constants with *p*_1_+*p*_2_+*p*_3_ = 1. Suppose that initially, for every node *v*, define the strategy of *v*, written by *s*(*v*)[0], to be *C*, *D* and *Q* with probabilities *p*_1_, *p*_2_ and *p*_3_ respectively. Suppose that *s*(*v*)[*t*] are defined for all *v*. Then for every *v*, the payoff of *v* during step *t*, written by payoff(*v*)[*t*] is the total payoffs of *v* with all its neighbors during step *t*. At step *t* + 1, for every *v*, find uniformly and randomly a neighbor *u* of *v*, if payoff(*u*)[*t*] > payoff(*v*)[*t*], then with probability $\frac{P(u)[t]-P(v)[t]}{b∙\max\{d_{u},d_{v}\}}$, define *s*(*v*)[*t* + 1] = *s*(*u*)[*t*], otherwise, then set *s*(*v*)[+1] = *s*(*v*)[*t*]. Let *X* be a strategy. Then define *ρ*_*X*_[*t*] to be the fraction of nodes of *G* that share strategy *X* during step *t*.

We say that strategy *X* converges in the evolutionary prisoner’s dilemma games on *G*, if there exist a time step *t*_0_, a small constant *ε*, less than 0.05 say, and large constant *δ*_0_, greater than 0.9 say, such that, almost surely, for every *t* ≥ *t*_0_, ∣*ρ*_*X*_[*t*]−*δ*_0_∣ ≤ *ε* holds.

**Fig 2:**
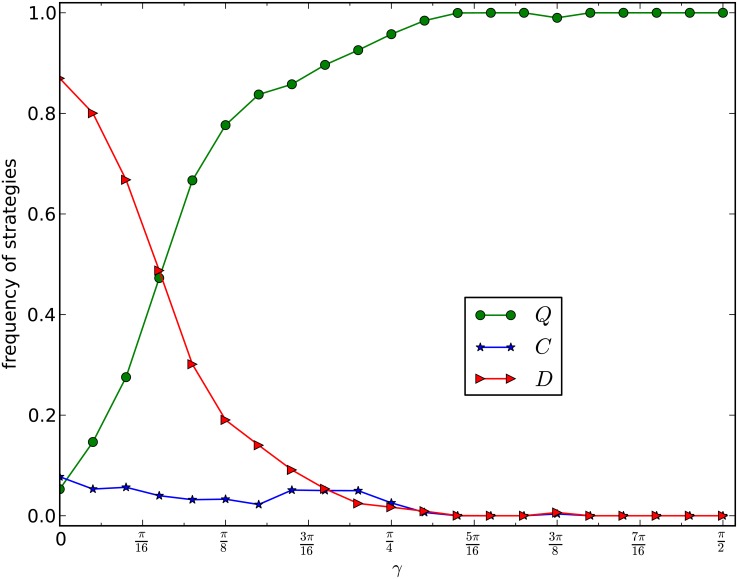
GPD_W_, PA model. The evolution of weak generalized prisoner’s dilemma games with time steps on a network generated by the PA model. Here the number of nodes of the graph is 10,000, the average number of edges of the graph is *d* = 4. The temptation factor of the game is *b* = 1.8, and the measurement of entanglement is $\gamma =\frac{\pi }{5}$. In this experiment, each of the *p*_*i*_ is chosen to be $\frac{1}{3}$. In the figure, qs, cs and ds are the curves of *ρ*_*Q*_, *ρ*_*C*_ and *ρ*_*D*_ respectively.

In Fig. 2, we depict the curves of *ρ*_*C*_[*t*], *ρ*_*D*_[*t*] and *ρ*_*Q*_[*t*] for the evolutionary generalized prisoner’s dilemma game on a network generated by the PA model.

From Fig. 2, we observe that:

1. After 2,000 steps, the *Q*-strategy nodes take more than 80% of the graph, and
2. After 3,000 steps, more than 90% or almost all nodes take the *Q*-strategy. This means that the *Q*-strategy is guaranteed to emerge quickly as the dominating strategy of the evolutions of the GPD’s on the power law network.

Fig. 2 shows the emergence of super cooperations for one set of parameters on one power law graph.

The experiment in Fig. 2 explores a general principle.

*Convergence of super cooperation principle*: For a fixed power law graph, and for a fixed temptation *b*, there is a small *γ*_0_ such that for every *γ* ≥ *γ*_0_, super cooperation of the evolution of the weak version of our generalized prisoner’s dilemma games on *G* with temptation *b* and *γ*, is almost surely guaranteed to quickly emerge and converge.

(Remark. We actually did a huge number of experiments, each of which verified this general principle.)

This progress also raises a question to theoretically prove the principle.

Therefore, the *Q*-strategy here plays a fundamental role in emergence and stochastic convergence of cooperations, and weak version of our generalized prisoner’s dilemma game is remarkably different from the classical weak prisoner’s dilemma game.

We notice that better convergence result holds for the normalised full version of our generalised prisoner’s dilemma games given in Table 3.

### Entanglement Eliminates Destroyers

To understand the reason why super cooperations quickly emerge in the evolution of the weak version of our generalized prisoner’s dilemma games on power law networks, we investigate the role of *γ* in the evolutions as *b* increases.

**Fig 3:**
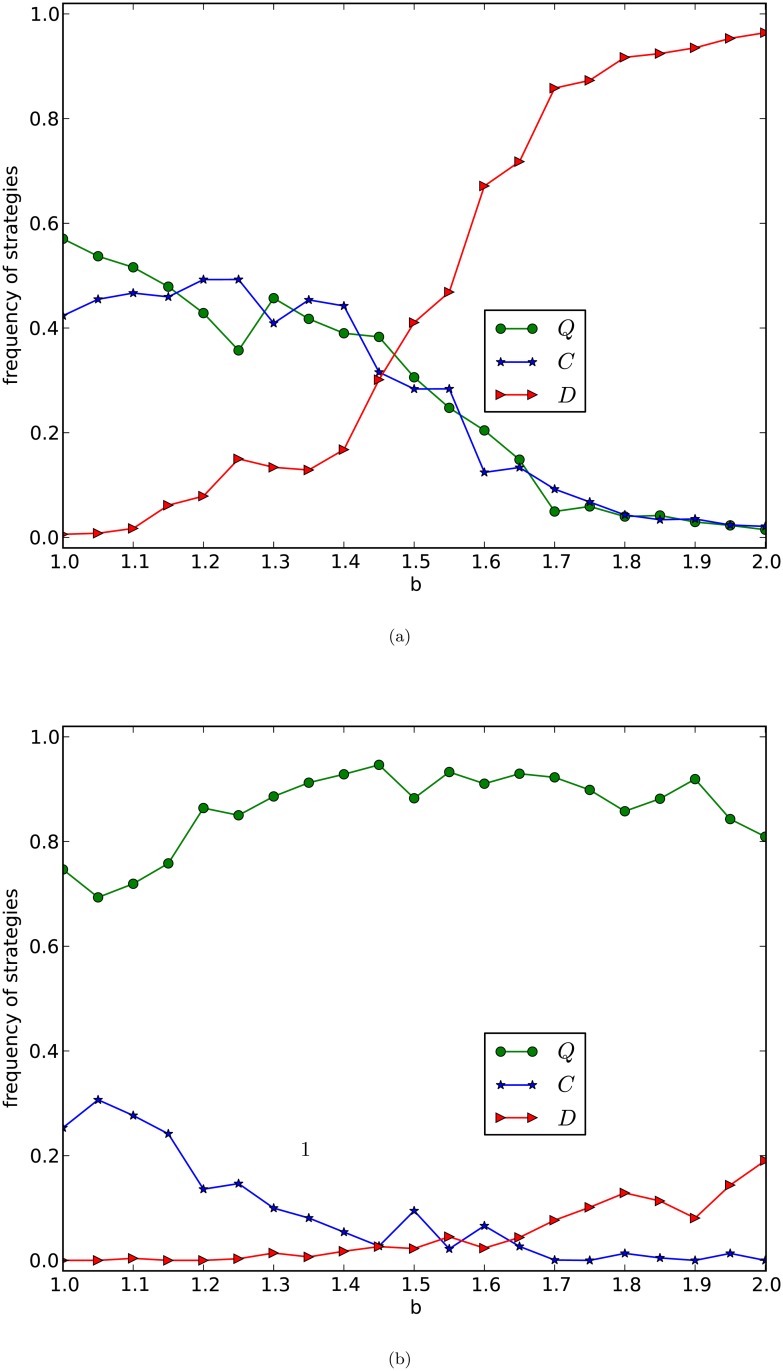
GPD_W_, PA model. The equilibrium frequencies of *Q*-, *C*- and *D*-strategies are plotted as functions of *b*. Part (a) and part (b) of the figures correspond to entanglement measurements *γ* = 0 and *γ* = *π*/6 respectively. The two parts give a comparison of the classical and generalized prisoner’s dilemma games. The network in this experiment has 10,000 nodes, and average number of edges *d* = 4. Each curve is the average frequencies of the last 5,000 rounds of 20,000 rounds games of 10 evolutions of 100 networks of the same type.

We depict, in Fig. 3, the curves of *ρ*_*Q*_, *ρ*_*C*_ and *ρ*_*D*_ as functions of *b* on a network generated by the PA model with number of nodes *n* = 10,000, and average number of edges *d* = 4. The curves in Fig. 3 are the average frequencies of the last 5,000 rounds of one evolution of 20,000 rounds of games for 10 times of evolutions of 100 networks of the same type.

By observing Fig. 3, we have the following results:

1) From Fig. 3 (a), for *γ* = 0, if *b* ≥ 1.4, then the frequency of *D*-strategy nodes increases and dominates the network, and
2) From Fig. 3 (b), for $\gamma =\frac{\pi }{6}$, if *b* ≤ 1.3, then *D* varnishes, and both *C* and *Q* coexist, and if *b* > 1.3, then *Q* quickly becomes the dominating strategy, in either case, the *D*-strategy nodes varnish from the network.

This experiment demonstrates that in the classic weak prisoner’s dilemma games, *D*-strategy nodes exist and may dominate the network, and that for the weak version of our generalized prisoner’s dilemma games, if *γ* is not too small, then the *D*-strategy nodes almost surely varnish, in which case, either *Q* becomes the dominating strategy or both *C* and *Q* coexist and share the network.

Therefore in any case, we have the following:

*Destroyer elimination principle*: The entanglement measure *γ* > 0 eliminates the destroyers, i.e., the *D*-strategy nodes, unless *γ* is trivially small.

Clearly, the same principles holds for the game in Table 3.

### Emergence of Super Cooperation or Coexistence of Cooperation and Super Cooperation—the Weak Version

It was shown that cooperation of evolutionary classical weak prisoner’s dilemma games on networks of the PA model with small *d*, less than 10 say, is relatively easy to emerge, and that cooperation of evolutionary classical weak prisoner’s dilemma games on networks of the PA model with large *d*’s are unlikely to emerge. Does this phenomenon hold for our generalized prisoner’s dilemma games?

**Fig 4:**
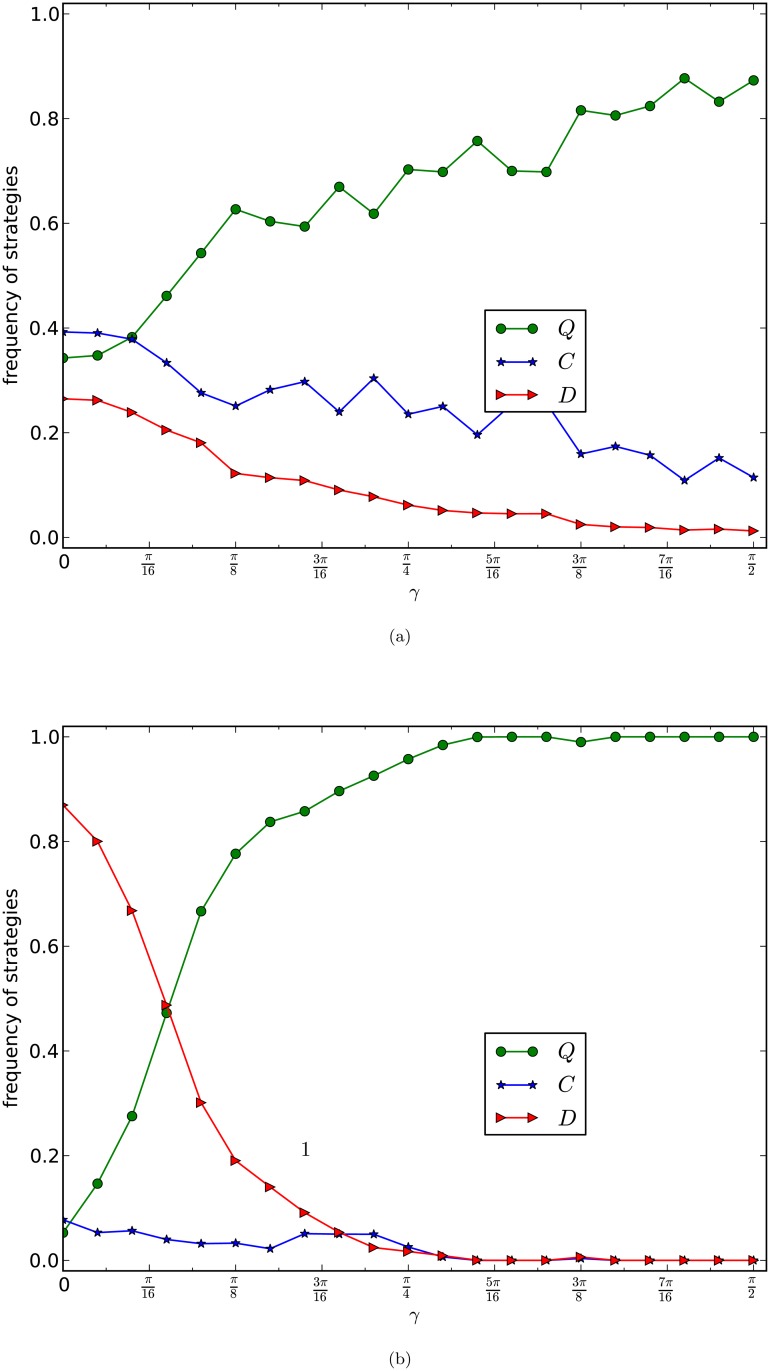
GPD_W_, PA model. The equilibrium frequencies of three strategies are plotted as a function of *γ* with *b* = 2. The size of the network is 10,000 and the average numbers of edges of the networks in (a) and (b) of the figure are 8 and 16 respectively. Each curve is the average frequencies of the last 5,000 rounds of 20,000 rounds games of 10 evolutions of 100 networks of the same type.

To answer this question, we investigate the equilibrium frequencies of strategies of our games on graphs generated by the PA model with different average numbers of edges. In Fig. 4 (a) and Fig. 4 (b), we depict the curves of the equilibrium frequencies of *Q*-, *C*- and *D*-strategies with fixed *b* = 2 as functions of *γ*, of evolutionary generalized prisoner’s dilemma games, given in Table 1, on networks of the PA model with number of nodes *n* = 10,000, and with average numbers of edges 8 and 16, respectively. The curves in Fig. 4 are the average frequencies of the last 5,000 rounds of one evolution of 20,000 rounds of games for 10 times of evolutions of 100 networks of the same type.

By observing Fig. 4 (a), we have the following results:

1) the equilibrium frequencies of the *D*-strategy nodes are always small, and decrease slowly,
2) the equilibrium frequencies of the *C*-strategy nodes are non-trivial and decrease slowly,
3) the equilibrium frequencies of the *Q*-strategy nodes are significantly large, increase slowly and reach a global fraction, around 80%, of nodes of the network, as *γ* increases, and
4) even if $\gamma =\frac{\pi }{2}$, the *Q*-strategy nodes fail to occupy the whole network, although *Q* is certainly the dominating strategy in the evolution.

1) and 2) imply that in this case, cooperation nontrivially exists in the evolution of the games for all *γ*’s. 2) and 3) imply that if *γ* is small, then the union of *C*- and *Q*-strategy nodes dominate the network, 3) and 4) imply that as *γ* increases, the *Q*-strategy nodes take more and more fraction of the nodes in the network during the evolution. 3) and 4) imply that even if $\gamma >\arccos\frac{\sqrt{b}}{b}$, *Q*-strategy nodes fail to occupy the whole network, although they must dominate the network. This gives rise to a difference between a local game between two players and the evolution of the games on a scale-free network of the PA model.

By observing Fig. 4 (b), we have the following results:

(1) the equilibrium frequencies of the *C*-strategy nodes are trivially small for all *γ*,
(2) if *γ* is small, then the equilibrium frequency of the *D*-strategy nodes dominates the network,
(3) as *γ* increases, the equilibrium frequency of the *D*-strategy nodes quickly decreases,
(4) the equilibrium frequency of the *Q*-strategy nodes quickly increases, as *γ* increases, and
(5) if *γ* is large enough, $\gamma >\arccos\frac{\sqrt{b}}{b}$ say, then the *Q*-strategy nodes certainly dominate or even occupy the whole network.

(1) means that if *d* is large, then *C*-strategy nodes are unlikely to survive. (2) and (3) mean that if *d* is large and *γ* is too small, then destroyers, the *D*-strategy nodes, must dominate the network. (4) means that the equilibrium frequencies of the *Q*-strategy nodes are almost linearly increasing as *γ* increases. (4) and (5) mean that if *γ* is appropriately large, then even if *d*’s are large, the *Q*-strategy nodes dominate or even occupy the network.

By comparing Fig. 4 (a) and Fig. 4 (b), we have:

(i) the curves of the frequencies of the *Q*-strategy nodes increase as *γ* increases, regardless of whether *d* is small or large,
(ii) if *d* is small, then there are always some *C*-strategy nodes,
(iii) if *d* is large, then *C*-strategy nodes are hard to survive,
(iv) the curves of frequencies of the *D*-strategy nodes are dramatically different for different *d*’s,
(v) in the case of *d* small, if *γ* is small, then the *C*- and *Q*-strategy nodes coexist and the union of the *C*- and *Q*-strategy nodes dominates the network, and if *γ* is large, then the *Q*-strategy nodes must dominate the network, and
(vi) in the case of *d* large, the *Q*-strategy nodes fight with the destroyers, in which case, the *Q*-strategy nodes are almost surely guaranteed to dominate the network if and only if *γ* is large, greater than $\arccos\frac{\sqrt{b}}{b}$ say.

The results above explore the following new principles.

*Emergence of super cooperation principle*: If $\gamma >\arccos\frac{\sqrt{b}}{b}$, then for networks of the PA model with arbitrarily given *d*, super cooperation is almost surely guaranteed to quickly emerge and converge in evolutionary generalized prisoner’s dilemma games given in Table 1 on the networks, but is unlikely to occupy the whole networks.

This means that, in the case of $\gamma >\arccos\frac{\sqrt{b}}{b}$, super cooperation is almost surely guaranteed to emerge and to keep forever after emerged on scale-free networks, regardless how complex the networks are.

*Coexistence of cooperation and super cooperation principle*: For networks of the PA model with small *d*, if *γ* is appropriately large, not necessarily greater than $\arccos\frac{\sqrt{b}}{b}$, then cooperation and super cooperation coexist and the union of cooperation (the *C*-strategy nodes) and super cooperation (the *Q*-strategy nodes) is almost surely guaranteed to quickly emerge in evolution of the weak version of our generalized prisoner’s dilemma games in Table 1 on the networks.

This means that in the case of $\gamma <\arccos\frac{\sqrt{b}}{b}$, if a scale-free network has some nice structure, then classic weak PD games allow some cooperators to survive, in which case, for the weak generalized prisoner’s dilemma games, supper cooperators coexist with some cooperators, and super cooperators and cooperators together dominate the network, unless *γ* is trivially small.

This principle explores that entanglement plays an essential role in emergence of super cooperation cooperation, that it is possible for cooperators and super cooperators coexist in a rich-get-richer world, even if they are different, and actually the two together dominate the networks, and that cooperator and super cooperation coexist in evolution of the weak version of our generalised prisoner’s dilemma games on a scale-free network only if the scale-free network has a community structure, in the case that the *d*’s are small.

### Generalised Prisoner’s Dilemma—Normalised Full Version

In this section, we investigate the equilibrium frequencies of the normalised full version of our generalised prisoner’s dilemma games given in Table 3, on scale-free networks of the PA model.

During the games, the updating probability of a node to adopt the strategy of a reference node, is determined by the Fermi function as follows. For each node *i*, *i* randomly and uniformly picks a neighbor *j*. Then the probability that node *i* adopts the last strategy of node *j* is defined by

$$P=\frac{1}{1+\exp[-\left(P_{j}-P_{i}\right)/T]},$$

where *P*_*i*_, *P*_*j*_ are the current total payoffs of nodes *i* and *j* respectively, and *T* is a parameter representing the noise of the updating strategy. In our experiments, we set *T* = 0.04.

**Fig 5:**
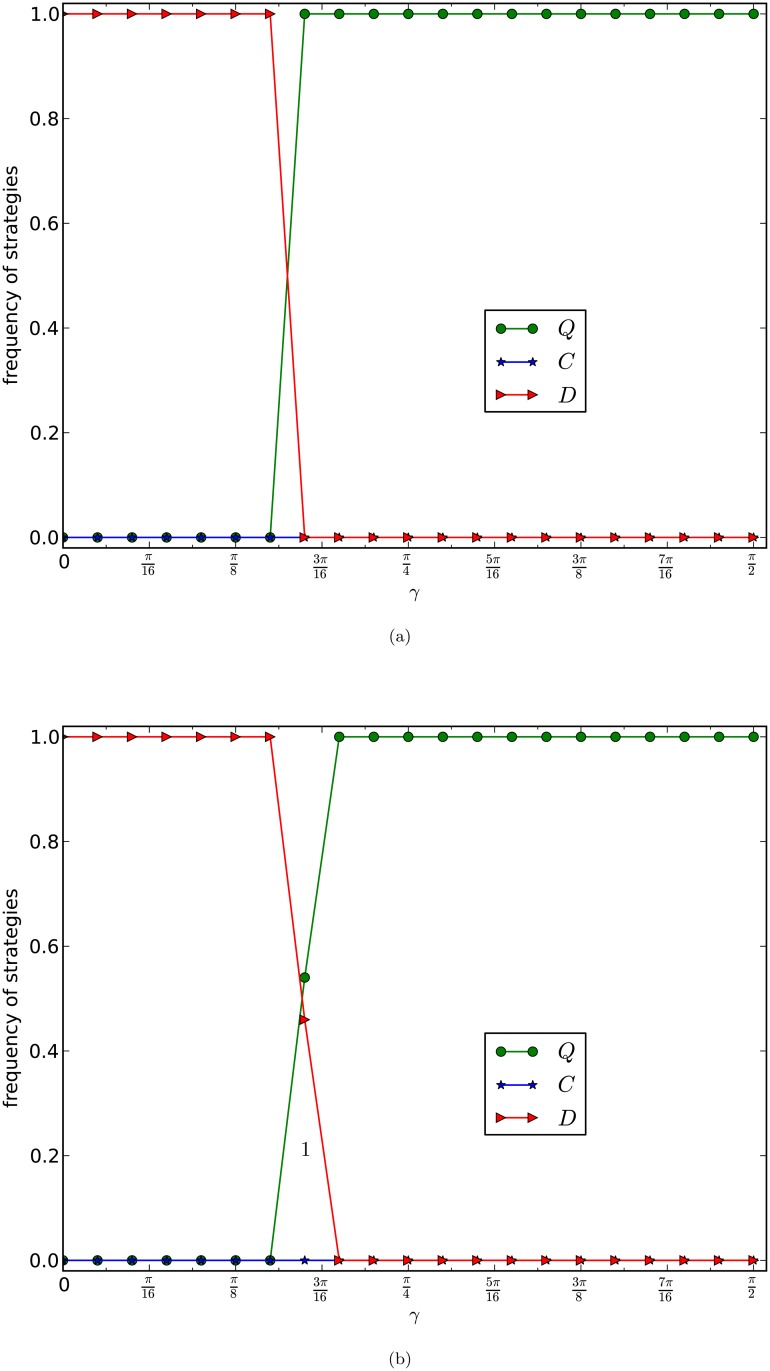
GPD_N_, PA model. The equilibrium frequencies of three strategies of the normalised prisoner’s dilemma games are plotted as a function of *γ* with *r* = 0.8. The size of the network is 10,000 and the average numbers of edges of the networks in (a) and (b) of the figure are 4 and 8 respectively. Each curve is the average frequencies of the last 5,000 rounds of 20,000 rounds games of 10 evolutions of 10 networks of the same type.

In Figs. 5 (a) and 5 (b), we depict the equilibrium frequencies of the three strategies of generalised true prisoner’s dilemma games on networks of the PA model with *d* = 4 and 8 respectively. The payoff matrix of the games in Fig. 5 is given in Table 3 with *r* = 0.8. The curves in Fig. 5 are the average frequencies of the last 5,000 rounds of one evolution of 20,000 rounds of games for 10 times of evolutions of 10 networks of the same type.

By observing Fig. 5, we have the following results:

(1) For *d* = 4, from Fig. 5 (a), there is a *γ*_0_ around the middle point between $\frac{2\pi }{16}$ and $\frac{3\pi }{16}$ such that if *γ* is slightly less than *γ*_0_, then the *D* strategy nodes occupy the network, and if *γ* is slightly larger than *γ*_0_, then the super cooperators, ie., the *Q*-strategy nodes, occupy the network.
(2) For *d* = 8, from Fig. 5 (b), there is a constant *γ*_0_ which is less than and close to $\frac{3\pi }{16}$ such that for any *γ*, if *γ* is slightly less than *γ*_0_, then the defectors occupy the network, and if *γ* is slightly larger than *γ*_0_, then the super cooperators occupy the network.
(3) From both (a) and (b) of the figure, we observe a *phase transition phenomenon* from the domination of defectors to the domination of super cooperators of the normalised full version of our generalised prisoner’s dilemma games on the scale-free networks, that there is a small interval (*γ*_0_, *γ*_1_) such that if *γ* ≤ *γ*_0_, then defectors occupy almost the whole network, and if *γ* ≥ *γ*_1_, then the super cooperators occupy almost the whole network.

By comparing Figs. 4 and 5, we observe the following remarkable differences:

- In the true prisoner’s dilemma game, the cooperator gets a worse position in a game with a defector than that in the weak prisoner’s dilemma game. Therefore in our generalised prisoner’s dilemma games, the cooperators are simply out of the game, so the competition becomes between the defectors and the super cooperators, for which the entanglement degree *γ* becomes crucial and critical with a sharp threshold.
- In the weak prisoner’s dilemma games, as we have already known that some times, cooperators may emerge. Therefore, if *γ* is large, then super cooperators dominate the network with certainty, otherwise, if *γ* is not too small, then cooperators and super cooperators coexist, and together dominate the network.
- The experiments show again that in the classic prisoner’s dilemma games, heterogeneity promotes emergence of cooperation only if the games are the weak version of the PD games, instead of the full version or normalised full version of the PD games.
- Our experiments also show that the updating strategy by the Fermi function (Fig. 5) and the updating strategy by linearity of the difference of payoffs (Figs. 1, 2, 3 and 4) play the same role in the evolution of the games.

### Normalised Payoff Updating Strategy

In this section, we implement experiments of the weak version of our generalised prisoner’s dilemma game in Table 1, by applying the normalised updating strategy introduced by Wu, Guan, Xu and Wang [18, 20]. It is a normalised version of the Fermi function, and proceeds as follows.

During the games, a node *i* randomly and uniformly picks a neighbor *j*. Then the probability that node *i* adopts the last strategy of node *j* is defined by

$$P=\frac{1}{1+\exp[-\left(\frac{P_{j}}{d_{j}}-\frac{P_{i}}{d_{i}}\right)/T]},$$

where *P*_*i*_, *P*_*j*_ are the current total payoffs of nodes *i* and *j* respectively, *d*_*i*_ and *d*_*j*_ are the degrees of nodes *i* and *j* respectively, and *T* is a parameter representing the noise of the updating strategy. Again, we set *T* = 0.04 in our experiments.

The normalised updating strategy is interesting due to the fact that in some cases, it reflects the human selection that a person may simply follow the strategy of the majority of his/her neighbors. This idea is supported by the human experiments in [35].

**Fig 6:**
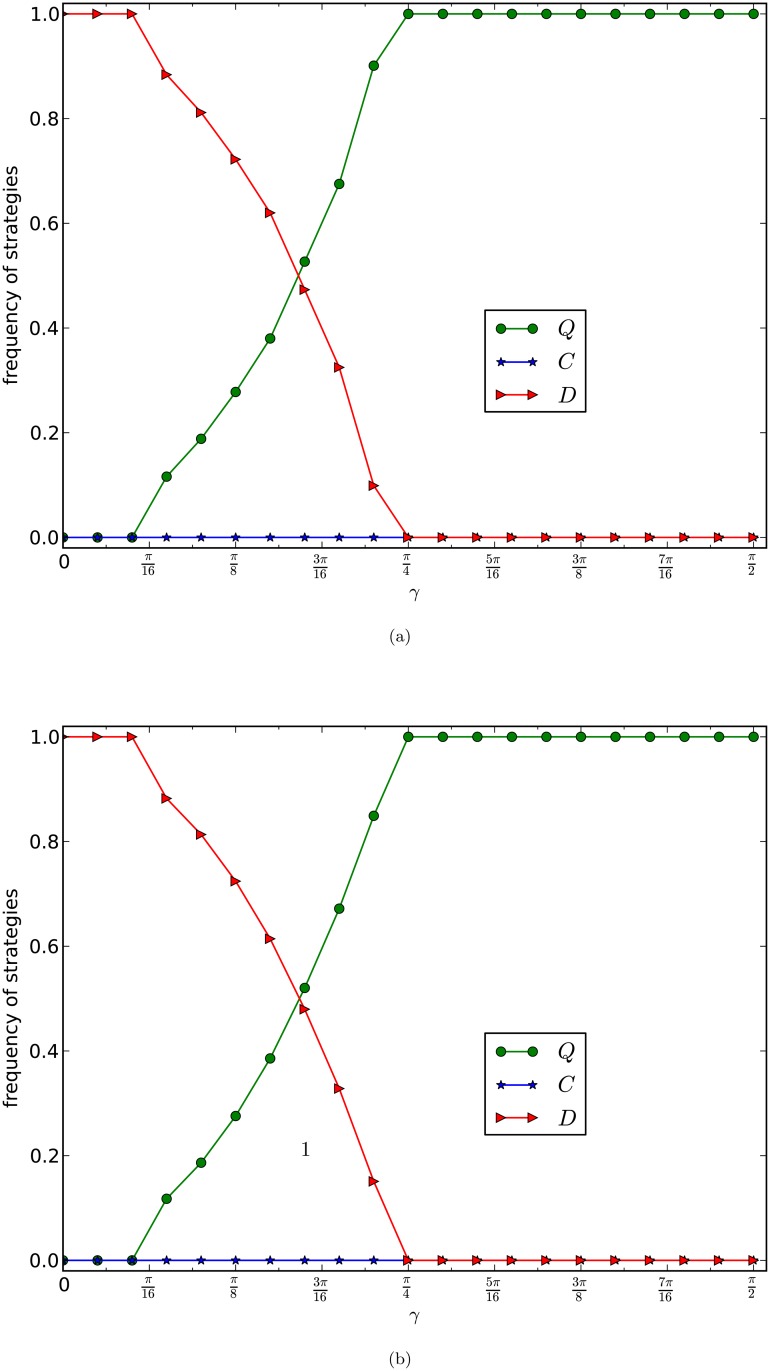
GPD_W_, PA model, normalised updating strategy. The equilibrium frequencies of three strategies of the weak prisoner’s dilemma game with normalised updating strategy are plotted as a function of *γ* with *b* = 2. The size of the network is 10,000 and the average numbers of edges of the networks in (a) and (b) of the figure are 4 and 8 respectively. Each curve is the average frequencies of the last 5,000 rounds of 20,000 rounds games of 10 evolutions of 10 networks of the same type.

In Fig. 6, we depict the equilibrium frequencies of the three strategies of the weak version of our generalised prisoner’s dilemma games on networks of the PA model. The curves in Fig. 6 are the average frequencies of the last 5,000 rounds of one evolution of 20,000 rounds of games for 10 times of evolutions of 10 networks of the same type.

By observing Fig. 6, and by comparing Fig. 6 with Figs. 5 and 4, we have the following results.

- Different from Fig. 4, and the same as that in Fig. 5, we have that the cooperators are simply out of the games. So the competition becomes the game between the defectors and the super cooperators. This supports the discovery in [18, 20] that cooperation fails to emerge in the evolutions of the weak version of PD games on scale-free networks.
- Similar to Fig. 5. There is a relatively large interval (*γ*_0_, *γ*_1_) such that
  - if *γ* ≤ *γ*_0_, then the defectors occupy the network,
  - if *γ* ≥ *γ*_1_, then the super cooperators occupy the network
  - in the interval (*γ*_0_, *γ*_1_), the super cooperators linearly increase, and the defectors linearly decreases
- Different from Fig. 5. The phase transition from the domination of defectors to the domination of super cooperators allows a wider range of the entanglement degree *γ*. That is, the interval in B. above is much larger than that in Fig. 5 for the normalised full version of our generalised prisoner’s dilemma game.

The results imply that normalised updating strategy actually homogenises the scale-free networks of the PA model so that the games in the scale-free networks behave as that in homogenous graphs, referred to as [32].

### Full Version of Our Generalised Prisoner’s Dilemma Game with Normalised Updating Strategy

**Fig 7:**
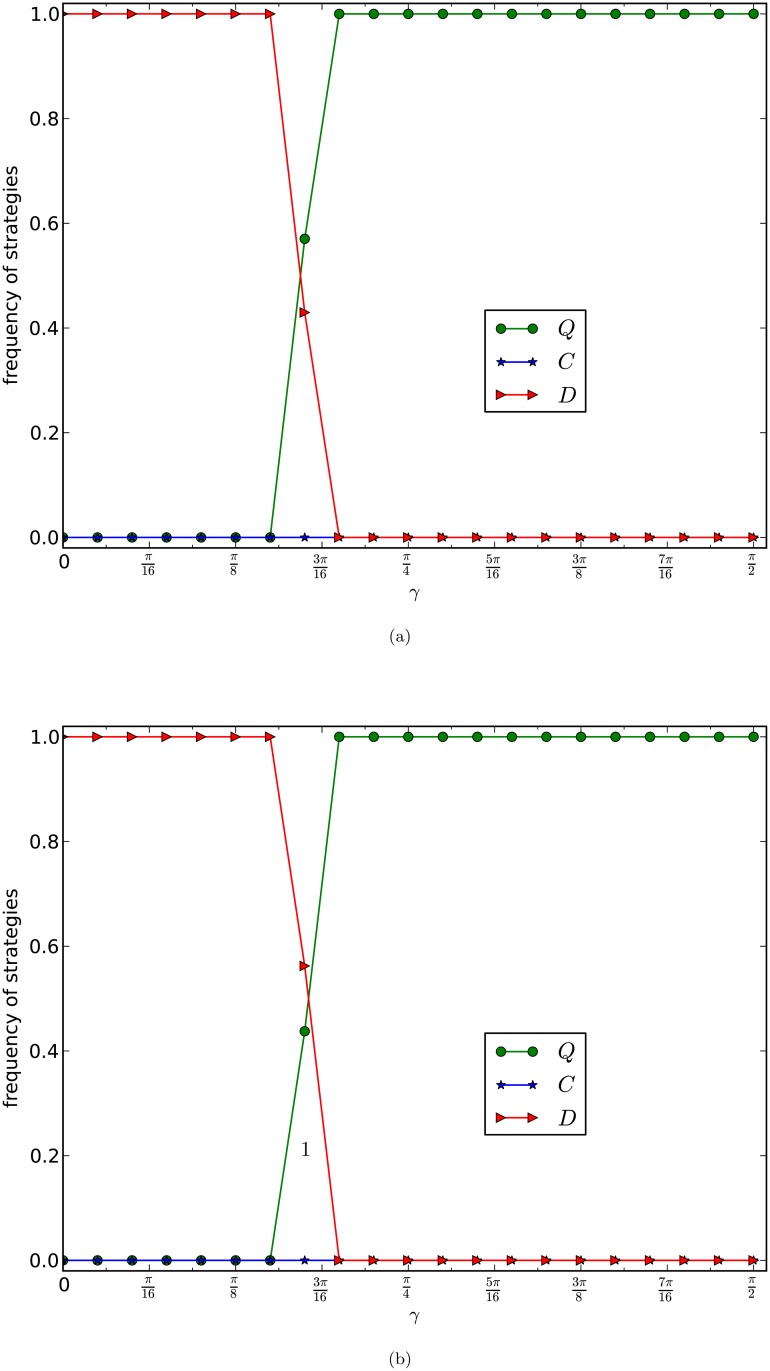
GPD_N_, PA model, normalised updating strategy. The equilibrium frequencies of three strategies of the normalised prisoner’s dilemma games with normalised updating strategy and with *r* = 0.8 are plotted as a function of *γ*. The size of the network is 10,000 and the average numbers of edges of the networks in (a) and (b) of the figure are 4 and 8 respectively. Each curve is the average frequencies of the last 5,000 rounds of 20,000 rounds games of 10 evolutions of 10 networks of the same type.

In Fig. 7, we implemented experiments of the normalised full version of our generalised prisoner’s dilemma games with the normalised updating strategy on scale-free networks of the PA model. The curves in Fig. 7 are the average frequencies of the last 5,000 rounds of one evolution of 20,000 rounds of games for 10 times of evolutions of 10 networks of the same type.

By observing Fig. 7 and by comparing Fig. 7 with Figs. 4, 5, 6, we have the following results:

(a) Fig. 7 is nearly as the same as Fig. 5 for the normalised full version of our generalised prisoner’s dilemma games.
(b) This implies that the game itself is more crucial to the emergence of cooperation than the updating strategies.
(c) The normalised updating strategy plays a role only for the weak version of the prisoner’s dilemma games, in both the classic and our generalised cases.

### Emergence of Super Cooperation Is Independent of Structures

Our results observed from Figs. 2–7 demonstrate that if the entanglement degree *γ* is appropriately large, then super cooperation is guaranteed to quickly emerge and to keep forever after emerged in the evolutions of the weak and normalised generalised prisoner’s dilemma games on scale-free networks with various updating strategies, and that if *γ* is not large enough, but not too small, then cooperators and super cooperators coexist and together dominate the network in the evolutions of weak generalised prisoner’s dilemma games with updating strategies defined by either linearity of payoffs or the Fermi function (but not by the normalised payoff strategy). Our results in [32] showed that for appropriately large *γ*, super cooperation is guaranteed to quickly emerge and to keep forever after emerged in the evolutions of the weak prisoner’s dilemma games on random graphs.

These results imply that super cooperation is guaranteed to quickly emerge and to keep forever after emerged in the evolutions of our generalised prisoner’s dilemma games on an arbitrarily given network, and that the convergence of super cooperation is guaranteed by appropriately large entanglement degree, which is independent of the structures of networks, and independent of the updating strategies of the games, and that in both classic and our generalised games, heterogeneity promotes cooperation only if the games are the weak version of the PD games, and the updating strategy is not normalised.

We thus conclude that entanglement is a mechanism for guaranteed emergence of super cooperation of evolutionary generalised prisoner’s dilemma games, that entanglement degree *γ* determines a phase transition from the domination of defectors to the domination of super cooperators, leaving an interval for competition between the defectors and super cooperators, and that the role of heterogeneity is very limited in both classic and generalised PD games.

Thus entanglement is a new rule for cooperation. However, entanglement is a simplified measure of a complicated relationship between two players, which could be the mixture of many factors including: kinship, willing of cooperation, worries of cheated, worries to be punished in the future, fairness etc. Nevertheless, the two players in a real world game always have a complicated, and entangled relationship. Our results showed that the entanglement helps the two players to cooperate. On the other hand, if the two players are independent of each other, then both may easily and simply defect.

## Conclusions and Discussion

We proposed three generalized prisoner’s dilemma (GPD) games by introducing entanglement between the players, and by introducing the strategy of super cooperator. The first game is a weak version based on the spatial game by Nowak and May, the second is the generalisation of the full prisoner’s dilemma game, and the third is the generalised prisoner’s dilemma game based on the normalised prisoner’s dilemma game. The games are natural generalizations of the classical prisoner’s dilemma game. Our games allow us to introduce the entangled relationship between the two players, which occurs in real world games. The generalized prisoner’s dilemma games satisfy new Nash equilibrium principles. We implemented various experiments of our games on scale-free networks of the PA model. From the experiments, we found that entanglement is the mechanism of convergence (guaranteed emergence) of (super) cooperation of evolutionary prisoner’s dilemma games on scale-free networks, that if the entanglement *γ* is appropriately large, then super cooperation is always guaranteed to quickly emerge, and keep forever after emerged, that if the entanglement degree *γ* is nontrivially large, then cooperators and super cooperators coexist and together dominate the network in the weak version of our generalised prisoner’s dilemma games, that there is a phase transition from defection to super cooperation for the normalised prisoner’s dilemma games on the scale-free networks of the PA model, and that the normalised updating strategy promotes super cooperation for the weak version generalised prisoner’s dilemma games. Our games and principles explore that entanglement is the mechanism of emergence and convergence of super cooperation in a rich-get-richer world, and that appropriate entanglement degree ensures that cooperation and super cooperation coexist and together dominate the scale-free networks with well-defined structures. Our generalized prisoner’s dilemma games explain the reason why a win-win cooperation occurs in a real world game. Our coexistence principle demonstrates that cooperators and super cooperators coexist and dominate the scale-free networks of well-defined structures, as taught by Confucius, the Chinese philosopher and teacher (551–479, BC), that honest people coexist in a well-built society even if they are different. Our theory demonstrates that entanglement is the mixture of the complicated, entangled, and even undefinable relationships among the players and that the entanglement becomes an essential resource for cooperations in evolutionary games. This progress also poses a number of fundamental issues such as: Is entanglement a well-defined measure of relationship between individuals in nature and society? Can we explain the evolutions of the nature and species by our theory? Can we apply our theory to solve some problems in the 21st century (international) economy and world trade?

## Methods

The curves in Figs. 3 and 4 are the average frequencies of the last 5,000 rounds of the evolutions of 20,000 rounds of 10 times evolutions of 100 networks of the same type. The curves in Figs. 5, 6, and 7 are the average frequencies of the last 5,000 rounds of the evolutions of 20,000 rounds of 10 times evolutions of 10 networks of the same type.
